# Relationship Between Sleep and Function in Older Adults With Mild Cognitive Impairment

**DOI:** 10.1093/geroni/igaa057.2104

**Published:** 2020-12-16

**Authors:** Miranda McPhillips, Junxin Li, Darina Petrovsky, Nancy Hodgson

**Affiliations:** 1 University of Pennsylvania, Philadelphia, Pennsylvania, United States; 2 Johns Hopkins School of Nursing, Baltimore, Maryland, United States; 3 University of Pennsylvania, Cherry Hill, New Jersey, United States

## Abstract

Our objective was to examine relationships between sleep characteristics and function in community-dwelling older adults with cognitive impairment. Sleep measures included actigraphy (total sleep time, wake after sleep onset, efficiency, awakenings), Pittsburgh Sleep Quality Index, Epworth Sleepiness Scale. Promis Physical Function Short Form and Promis Item Bank Social were used to measure physical function and social activity. We used Spearman’s correlation and multivariate linear regression. In bivariate analyses, physical function was significantly related to daytime sleepiness, wake after sleep onset and awakenings; social activity was significantly related to sleep quality, daytime sleepiness, total sleep time, wake after sleep onset and number of awakenings. Controlling for cognition and age, sleep quality was independently associated with physical function (β= -0.80; p= 0.002). Relationships between sleep and social activity did not remain significant in multivariate analyses. Preliminary results suggest subjective sleep quality is most related to physical function.

